# HMGB1 functional plasticity in neurodegenerative diseases and its translational implications

**DOI:** 10.3389/fimmu.2026.1928641

**Published:** 2026-09-29

**Authors:** Weicai Cai, Yaqiao Yi, Rong Zeng, Yitong Yin, Jiayue Zhao, Jieyang Zhang, Guilong Fu, Hui Xu

**Affiliations:** 1 School of Traditional Chinese Medicine, Hunan University of Chinese Medicine, Changsha, Hunan, China; 2 Department of Rehabilitation Medicine, People's Hospital of Ningxiang City, Hunan University of Chinese Medicine, Changsha, China; 3 School of Traditional Chinese Medicine, Hunan Traditional Chinese Medical College, Zhuzhou, Hunan, China

**Keywords:** damage-associated molecular patterns, glial-neuronal interactions, HMGB1, neurodegenerative diseases, neuroinflammation, redox regulation

## Abstract

High mobility group box 1 (HMGB1) is a chromatin-associated protein that can be redistributed and released during cellular stress, when it acts as an extracellular alarmin within the broader family of damage-associated molecular patterns (DAMPs). Its functions depend on subcellular localization, redox and post-translational modification state, cellular source, mode of release, and molecular partners. This critical narrative review examines how these variables shape HMGB1 biology in Alzheimer’s disease (AD), Parkinson’s disease (PD), amyotrophic lateral sclerosis (ALS), Huntington’s disease (HD), spinocerebellar ataxias (SCAs), frontotemporal dementia (FTD), and related disorders, and considers their implications for biomarker and therapeutic development. Intracellular HMGB1 contributes to chromatin organization, DNA repair, mitochondrial genome maintenance, and proteostasis. After redistribution and release, extracellular HMGB1 signals through receptors including the receptor for advanced glycation end products (RAGE) and Toll-like receptor 4 (TLR4), interacts with disease-associated proteins, and links proteostatic stress with glial activation, neuronal dysfunction, and blood-brain barrier impairment. Across disease models, two recurrent patterns emerge: loss or sequestration of intracellular HMGB1 in models of polyglutamine disorders, including HD and SCAs, and extracellular HMGB1 signaling as a progression-associated inflammatory amplifier in AD, PD, and ALS models. Central-peripheral inflammatory communication and cell-specific HMGB1 release further shape these responses. Clinical studies have associated total HMGB1 or anti-HMGB1 autoantibodies with clinical features, but current assays do not resolve redox form, cellular source, or biological activity. Most therapeutic studies remain preclinical, while available human intervention studies involve drugs with multiple molecular actions. Clinical translation requires redox-resolved and standardized assays, longitudinal human sampling, compartment- and cell-specific causal studies, and interventions with documented target engagement, central nervous system exposure, and long-term safety. HMGB1 is best understood as a context-dependent mediator rather than a uniformly pathogenic factor or a single treatment target.

## 1 Introduction

Population ageing is increasing the global burden of neurodegenerative diseases. In 2021, conditions affecting the nervous system were the leading source of health loss worldwide, with Alzheimer’s disease (AD) and other dementias among the largest contributors in older adults (1). The global number of people living with dementia was estimated at 57.4 million in 2019 and is projected to reach 152.8 million by 2050 (2). Although AD, Parkinson’s disease (PD), amyotrophic lateral sclerosis (ALS), and Huntington’s disease (HD) differ in etiology and regional vulnerability, they share several pathological features, including progressive neuronal dysfunction and loss, abnormal protein accumulation, impaired proteostasis, and sustained changes in glial and immune activity (3–5). HMGB1 connects several of these intracellular stress responses with extracellular inflammatory signaling.

High mobility group box 1 (HMGB1) is an evolutionarily conserved, non-histone chromatin-binding protein. Under homeostatic conditions, HMGB1 is predominantly nuclear and contributes to chromatin organization, transcriptional regulation, and DNA repair. Cellular stress and post-translational modifications can promote its translocation to the cytoplasm, followed by active secretion from immune or glial cells or passive release from cells that lose membrane integrity. Once extracellular, HMGB1 functions as an alarmin within the broader family of damage-associated molecular patterns (DAMPs). It signals through receptors that include the receptor for advanced glycation end products (RAGE) and Toll-like receptor 4 (TLR4), while its receptor usage and biological activity are influenced by redox state, post-translational modifications, and molecular binding partners (6–10).

This compartment-dependent functional plasticity places HMGB1 at the interface between neuronal homeostasis and neuroinflammation. Loss or sequestration of nuclear HMGB1 can disrupt chromatin regulation and DNA repair, whereas cytoplasmic HMGB1 participates in the regulation of autophagy (11, 12). Extracellular HMGB1 can activate microglia and astrocytes, alter inflammatory signaling, and contribute to blood-brain barrier dysfunction (13–15). HMGB1 also intersects with disease-associated proteins and proteostatic pathways involving amyloid-β, tau, α-synuclein, and polyglutamine-expanded proteins (11, 13, 14). Its effects therefore depend on subcellular localization, biochemical state, cellular source, and disease context.

Clinical studies have examined HMGB1 in blood and cerebrospinal fluid as a candidate marker of neuroinflammatory activity, while experimental studies have tested neutralizing antibodies, competitive Box A antagonists, glycyrrhizin, and modulators of HMGB1 release or receptor signaling (11, 14, 16–18). Together, these approaches extend HMGB1 research from mechanistic studies to biomarker development and therapeutic targeting.

Previous reviews have summarized the general biology of HMGB1, its involvement across neurodegenerative diseases, and its disease-specific roles in AD and PD (11, 13, 14, 16, 18, 19). This critical narrative review focuses on how subcellular localization, redox and post-translational modification state, cellular source, and disease stage shape HMGB1 function across AD, PD, ALS, HD, spinocerebellar ataxias, and frontotemporal dementia. It compares two recurrent patterns—loss of nuclear HMGB1 function and gain of extracellular inflammatory activity—and examines their implications for biomarker development and therapeutic targeting. The review first outlines the molecular basis of HMGB1 functional plasticity, then examines its disease-specific roles, and finally considers shared mechanistic themes, translational challenges, and research priorities.

## 2 Molecular determinants of HMGB1 functional plasticity

High-mobility group box 1 (HMGB1) was initially identified as a chromatin-associated protein and was subsequently recognized as an extracellular mediator released by activated macrophages and damaged cells (20, 21). These intracellular and extracellular activities arise from the same polypeptide but are governed by its structural state, post-translational modifications, subcellular localization, mode of release, and receptor context. Together, these variables shape the functional state of HMGB1 in disease-associated settings.

### 2.1 Structure, redox states, and post-translational modifications

Human HMGB1 is a 215-amino-acid protein of approximately 25 kDa. It contains two homologous DNA-binding domains, the A box (residues 9-79) and B box (residues 89-163), followed by an acidic C-terminal tail (residues 186-215) (22–24). Each HMG box adopts an L-shaped fold formed by three α-helices and binds the minor groove of DNA without sequence specificity. By bending DNA and increasing its local flexibility, the two boxes facilitate the assembly of chromatin-associated protein complexes (22, 23). The B box contains the principal cytokine-stimulating region of extracellular HMGB1 (25).

The C-terminal tail consists of a highly acidic intrinsically disordered region. Together with the flexible inter-box linker, it forms transient electrostatic contacts with the DNA-binding domains and regulates their accessibility to DNA and other binding partners (24, 26). These multivalent contacts produce a dynamic, autoinhibited ensemble rather than a single fixed conformation. The same conformational flexibility contributes to intermolecular interactions. For example, fully reduced HMGB1 forms a dynamic complex with C-X-C motif chemokine ligand 12 (CXCL12), in which both structured domains and the acidic tail participate in binding (27).

HMGB1 contains three conserved cysteine residues: Cys23 and Cys45 in the A box and Cys106 in the B box. Their oxidation state defines three extracellular isoforms—fully reduced HMGB1 (frHMGB1), disulfide HMGB1 (dsHMGB1), and terminally oxidized HMGB1 (oxHMGB1)—with distinct receptor interactions and biological activities (28, 29). Their cysteine configurations and principal extracellular functions are summarized in **Table 1**.

**Table 1:** Redox isoforms and principal extracellular activities of HMGB1.

HMGB1 isoform	Cysteine configuration	Principal receptor or molecular partner	Main extracellular activity	Functional interpretation	Ref.
Fully reduced HMGB1 (frHMGB1; all-thiol HMGB1)	Cys23, Cys45, and Cys106 remain in the reduced thiol state	Forms a heterocomplex with C-X-C motif chemokine ligand 12 (CXCL12), which signals through C-X-C chemokine receptor type 4 (CXCR4)	Promotes chemotaxis and cell recruitment	Chemotactic form of extracellular HMGB1	(29, 30)
Disulfide HMGB1 (dsHMGB1)	An intramolecular disulfide bond forms between Cys23 and Cys45, while Cys106 remains reduced	Toll-like receptor 4 (TLR4)-myeloid differentiation factor 2 (MD-2) complex	Induces pro-inflammatory cytokine production	Cytokine-inducing form of extracellular HMGB1	(29, 31)
Terminally oxidized HMGB1 (oxHMGB1)	Cysteine residues undergo terminal oxidation to sulfonates	No receptor is assigned to its canonical chemotactic or cytokine-inducing activities	Lacks the chemotactic activity of frHMGB1 and the cytokine-inducing activity of dsHMGB1	Functionally inactive with respect to these two canonical extracellular activities	(29, 32)

Beyond redox regulation, acetylation, phosphorylation, and S-nitrosylation regulate HMGB1 trafficking and release. HMGB1 contains two nuclear localization signals, NLS1 within residues 28–44 and NLS2 within residues 179-185. Hyperacetylation of lysine residues within these regions shifts HMGB1 toward the cytoplasm, where it can enter secretory lysosomes (33). Phosphorylation within the NLS regions reduces binding to the nuclear import receptor karyopherin-α1, inhibits nuclear re-entry, and redirects HMGB1 toward secretion (34). S-nitrosylation of HMGB1 at Cys106 also promotes its nucleocytoplasmic translocation and active secretion from stimulated macrophages and microglia (35).

These modifications act at different stages of HMGB1 mobilization. Acetylation, phosphorylation, and S-nitrosylation contribute primarily to nucleocytoplasmic redistribution and release, whereas the frHMGB1, dsHMGB1, and oxHMGB1 configurations specify major extracellular activities. Cytoplasmic translocation alone does not determine the redox state or receptor activity of released HMGB1.

### 2.2 Compartment-specific functions and release mechanisms

Under basal conditions, HMGB1 is concentrated in the nucleus, where it functions as an architectural DNA-binding protein. Its transient association with DNA promotes DNA bending, nucleosome dynamics, and access of transcriptional and repair complexes to chromatin (22, 23, 36–39). HMGB1 also recognizes distorted and damaged DNA structures and participates in nucleotide excision repair, base excision repair, double-strand break repair, and other genome-maintenance processes (36, 39). It therefore acts as a DNA chaperone and transcriptional co-regulator rather than as a sequence-specific transcription factor (37, 38).

Following cellular stress or activation, HMGB1 can accumulate in the cytoplasm. In addition to serving as an intermediate pool for secretion, cytoplasmic HMGB1 participates in autophagy regulation. It binds Beclin 1 (BECN1), displaces B-cell lymphoma 2 (BCL2) from the BECN1-BCL2 complex, and promotes autophagic flux (12). The cytoplasmic pool is therefore functionally distinct from both chromatin-associated HMGB1 and extracellular receptor-active HMGB1.

HMGB1 lacks an N-terminal signal peptide and is not secreted through the conventional endoplasmic reticulum-Golgi pathway. Activated monocytes, macrophages, and microglia release HMGB1 through unconventional pathways involving cytoplasmic translocation, vesicular trafficking, secretory lysosomes, and secretory autophagy (33, 35, 40, 41). Active secretion is regulated by post-translational modification and requires intracellular trafficking before membrane export. HMGB1 is also passively released when lytic cell death disrupts plasma membrane integrity (40). Passive release does not require prior nuclear export and can discharge nuclear and cytoplasmic HMGB1 together with other intracellular components.

Once released, HMGB1 functions as an alarmin, a class of dual-function proteins within the broader category of damage-associated molecular patterns (DAMPs) (8, 40). Its extracellular activity depends on cysteine redox state, molecular partners, and receptor availability in recipient cells; accordingly, extracellular HMGB1 occurs as frHMGB1, dsHMGB1, oxHMGB1, or HMGB1-containing complexes.

### 2.3 Cell sources, receptors, and the working conceptual framework

Within the central nervous system, neurons, microglia, and astrocytes can serve as sources and targets of extracellular HMGB1 (19, 42, 43). Neuronal injury or lytic death can release intracellular HMGB1, whereas activated glial cells can secrete it through regulated pathways. The mode of release influences the accompanying intracellular material and the biochemical environment encountered by HMGB1 after release. Receptor expression on neurons, glia, endothelial cells, and infiltrating immune cells then determines which extracellular signals are transmitted.

Three receptor systems are particularly relevant to the extracellular functions of HMGB1. First, dsHMGB1 binds the TLR4-MD-2 complex through a configuration that requires reduced Cys106. This interaction activates myeloid differentiation primary response 88 (MyD88)-dependent signaling and nuclear factor-κB, leading to cytokine production (29, 31). Second, frHMGB1 forms a heterocomplex with CXCL12 and enhances CXCR4-dependent cell migration (27, 29, 30). Third, the receptor for advanced glycation end products (RAGE) binds extracellular HMGB1 either alone or in complexes with nucleic acids and other ligands. RAGE contributes to HMGB1 uptake and to sustained mitogen-activated protein kinase and nuclear factor-κB signaling in receptor-expressing cells (8–10, 44). Unlike TLR4-MD-2 and CXCR4, RAGE has not been restricted to a single HMGB1 redox isoform.

These receptor routes produce distinct outputs. The dsHMGB1-TLR4-MD-2 axis primarily induces inflammatory cytokine production, the frHMGB1-CXCL12-CXCR4 complex promotes chemotaxis, and RAGE supports context-dependent signaling and internalization of HMGB1-containing complexes. The extracellular response therefore depends on both the biochemical form of HMGB1 and the receptor repertoire of the target cell.

Accordingly, HMGB1 function can be described through four linked variables: its intracellular or extracellular compartment, its redox and post-translational modification state, the source cell and mode of release, and the receptor repertoire of the target cell. Nuclear HMGB1 denotes the chromatin-associated pool involved in genome maintenance; cytoplasmic HMGB1 denotes a translocated intracellular pool involved in autophagy regulation and secretion; and extracellular HMGB1 requires further specification as frHMGB1, dsHMGB1, oxHMGB1, or a molecular complex. Together, these variables define two recurrent HMGB1-associated processes in neurodegenerative disease: disruption of nuclear HMGB1 functions and amplification of extracellular inflammatory signaling.

The compartment-specific functions and stress-induced redistribution of HMGB1 are summarized in **Figure 1**.

**Figure 1:**
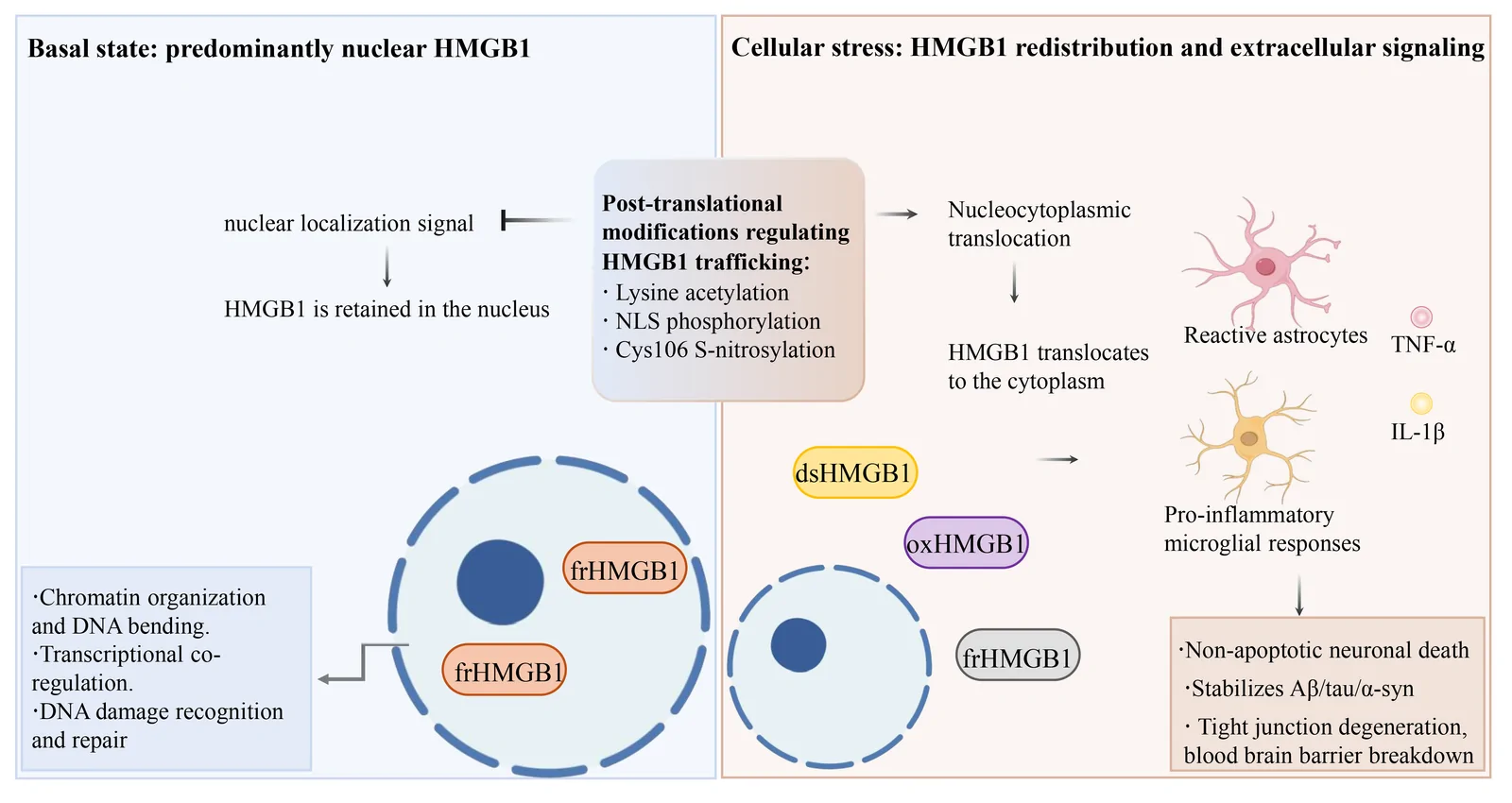
Compartment-dependent functions and stress-induced redistribution of HMGB1. Under basal conditions, HMGB1 is predominantly retained in the nucleus, where it contributes to chromatin organization and DNA damage repair. Lysine acetylation, phosphorylation within the nuclear localization signals, and S-nitrosylation at Cys106 promote its translocation to the cytoplasm. Following release, extracellular HMGB1 occurs in fully reduced, disulfide, and terminally oxidized forms, which differ in receptor engagement and biological activity. Extracellular HMGB1 participates in glial inflammatory responses and is associated with neuronal injury, pathological protein accumulation, and blood-brain barrier dysfunction in neurodegenerative disease. Aβ, amyloid-β; BBB, blood-brain barrier; dsHMGB1, disulfide HMGB1; frHMGB1, fully reduced HMGB1; HMGB1, high-mobility group box 1; IL-1β, interleukin-1β; NLS, nuclear localization signal; NLRP3, NLR family pyrin domain containing 3; oxHMGB1, terminally oxidized HMGB1; TNF-α, tumor necrosis factor-α; α-syn, α-synuclein.

## 3 HMGB1 in Alzheimer’s disease

Alzheimer’s disease (AD) is characterized by amyloid-β (Aβ) deposition, tau pathology, synaptic dysfunction, and progressive neuronal loss, accompanied by marked glial and neurovascular changes. HMGB1 intersects several of these processes through its effects on Aβ handling, tau-associated cellular stress, neuronal genome maintenance, and blood-brain barrier integrity. Its contribution varies with cellular source, subcellular localization, and the pathological environment.

### 3.1 Interactions with Aβ, tau, and proteostasis

Extracellular HMGB1 directly influences Aβ aggregation and clearance. HMGB1 binds Aβ1-42, stabilizes soluble Aβ oligomers, and colocalizes with Aβ in senile plaques in postmortem AD tissue (45). In cultured microglia, HMGB1 inhibits the uptake of Aβ1–42 and delays the degradation of Aβ1-40, thereby reducing microglial clearance of extracellular Aβ (46, 47). These effects connect extracellular HMGB1 with both the persistence of soluble Aβ assemblies and impaired plaque-associated clearance.

A recent study identified an additional link between HMGB1 and astrocytic proteostasis. Phosphoglycerate dehydrogenase (PHGDH), independently of its canonical metabolic activity, promoted the transcription of inhibitor of nuclear factor-κB kinase subunit α (IKKα) and HMGB1 in astrocytes. This transcriptional program suppressed autophagy and increased amyloid accumulation and synaptic injury in mouse models and human brain organoids (48). Thus, HMGB1 can participate in amyloid pathology through extracellular interactions with Aβ and through intracellular programs that impair autophagic clearance.

Tau pathology also engages HMGB1-dependent astrocytic responses. Tau oligomers induced the nucleocytoplasmic translocation and extracellular release of HMGB1 in primary astrocytes, accompanied by cellular senescence and a senescence-associated secretory phenotype. Pharmacological inhibition of HMGB1 release attenuated these changes and reduced tau oligomer and senescent-cell loads in hTau mice (49). This pathway links tau-associated cellular stress to persistent astrocytic dysfunction and inflammatory signaling.

Apolipoprotein E4 (APOE4) further modifies the relationship between tau pathology and HMGB1. In a tauopathy mouse model, neuronal APOE4 promoted HMGB1 acetylation, nucleocytoplasmic translocation, and release. Inhibiting HMGB1 release reduced gliosis, tau pathology, neurodegeneration, and myelin abnormalities (50). In a human study comprising 27 participants with mild cognitive impairment (MCI) and 25 cognitively normal controls, the interaction between APOE4 and plasma HMGB1 was associated with widespread cortical thinning (51). These findings place HMGB1 at the intersection of tau-associated neuronal stress, APOE4-dependent glial responses, and structural brain changes.

### 3.2 Glial responses, cell death, and blood-brain barrier dysfunction

HMGB1-associated glial responses in AD are closely coupled to local protein pathology. At amyloid deposits, extracellular HMGB1 reduces microglial uptake and degradation of Aβ (45–47). Tau oligomers stimulate HMGB1 release from astrocytes and promote cellular senescence (49), whereas APOE4 enhances neuronal HMGB1 release and the accompanying microglial and astrocytic responses in tauopathy models (50). These processes alter protein clearance, inflammatory signaling, and cellular homeostasis across neuronal and glial populations.

A series of studies by Okazawa and colleagues proposed that HMGB1 also participates in neuronal injury before extensive extracellular Aβ deposition. In this model, HMGB1 released from damaged neurons activates TLR4 signaling and increases phosphorylation of myristoylated alanine-rich C-kinase substrate (MARCKS) at Ser46, resulting in neurite degeneration. The authors designated this pre-aggregation interval as “phase 0” of AD pathology (52, 53). This model connects an early episode of neuronal injury with local HMGB1 signaling and subsequent disruption of neurites.

The same research program identified transcriptional repression-induced atypical cell death (TRIAD), a form of Yes-associated protein (YAP)-dependent neuronal necrosis, as another potential source and consequence of HMGB1 release. In AD mouse models and human induced pluripotent stem cell-derived neurons, intracellular Aβ sequestered YAP in the cytoplasm and impaired its nuclear transcriptional activity. Restoration of YAP activity reduced early neuronal necrosis and later amyloid pathology (54). HMGB1 released from affected neurons subsequently activated TLR4 and protein kinase C alpha (PKCα) signaling in neighboring neurons. PKCα-mediated phosphorylation of Ku70 at Ser77/78 impaired non-homologous end joining, increased DNA double-strand breaks, and induced secondary TRIAD-like neuronal injury (55). Increased Ku70 phosphorylation and DNA damage markers were also detected in postmortem AD brain tissue. TRIAD therefore represents one HMGB1-linked mechanism through which early neuronal injury could extend to neighboring cells.

HMGB1 also acts at the neurovascular interface. Serum profiling in cognitively normal individuals and patients with MCI or AD identified disease-associated changes in HMGB1 and soluble receptor for advanced glycation end products levels. In human brain microvascular endothelial monolayers, HMGB1 increased paracellular permeability and disrupted the distribution of the tight-junction protein zonula occludens-1; neutralization of HMGB1 attenuated these effects (15). These findings connect extracellular HMGB1 with loss of endothelial barrier integrity.

Peripheral inflammatory exposure can further alter HMGB1-associated responses in the AD brain. In 5xFAD mice, ozone exposure increased amyloid pathology and changed plaque-associated astrocyte profiles. Deletion of Hmgb1 from peripheral myeloid cells altered the induction of disease-associated astrocyte markers after ozone exposure (56). Peripheral myeloid HMGB1 is therefore a component of the lung-brain signaling response triggered by this environmental stressor.

### 3.3 Disease-specific mechanistic integration

HMGB1 integrates protein pathology with glial, neuronal, and neurovascular dysfunction in AD. Extracellular HMGB1 alters Aβ stability and clearance, while astrocytic PHGDH-dependent HMGB1 expression and tau- or APOE4-induced redistribution connect impaired proteostasis with cellular senescence and gliosis (45–51). Neuron-derived HMGB1 extends injury through TLR4-associated neurite degeneration and DNA-repair impairment in the phase 0 and TRIAD models (52–55). Endothelial and peripheral-myeloid responses further link this local signaling to blood-brain barrier dysfunction and ozone-associated lung-brain communication (15, 56).

## 4 HMGB1 in Parkinson’s disease

Parkinson’s disease (PD) is characterized by the progressive loss of dopaminergic neurons in the substantia nigra pars compacta and the accumulation of α-synuclein in Lewy pathology. In PD models and patient samples, changes in HMGB1 localization and release have been associated with glial activation, impaired α-synuclein clearance, and cellular stress. These processes place HMGB1 at the interface between dopaminergic neuronal injury, inflammatory signaling, and proteostasis.

### 4.1 Glial activation and inflammatory signaling

Extracellular HMGB1 contributes to sustained microglial activation in models of progressive dopaminergic neurodegeneration. Gao et al. showed that HMGB1 released from activated microglia and injured neurons bound macrophage antigen complex 1 (Mac1; CD11b/CD18) on microglia, activating NF-κB and NADPH oxidase and increasing superoxide production. HMGB1 neutralization, Mac1 deletion, or loss of the NADPH oxidase subunit gp91*phox* attenuated the progressive loss of dopaminergic neurons in neuron-glia cultures (57). This pathway provides a direct mechanism through which extracellular HMGB1 couples neuronal damage to a persistent microglial response.

RAGE and TLR4 are also involved in HMGB1-associated inflammation in PD models. Paraquat exposure increased HMGB1 expression and release and activated RAGE-dependent inflammatory signaling in microglia and the nigrostriatal system (58). In a study of 120 patients with PD and 100 healthy controls, serum HMGB1 and TLR4 levels were elevated in PD and increased with disease duration and Hoehn and Yahr stage. Serum NF-κB and TNF-α levels correlated with activation of the HMGB1-TLR4 axis (59). A separate study identified higher HMGB1 levels in the serum and cerebrospinal fluid of patients with PD and increased HMGB1 expression and cytoplasmic localization in postmortem substantia nigra tissue (60).

Post-translational modification further shapes the inflammatory activity of HMGB1 in PD-related settings. S-nitrosylation of HMGB1 at Cys106 by inducible nitric oxide synthase-derived nitric oxide promoted its nucleocytoplasmic translocation and secretion from activated microglia and macrophages. In neuron-glia cultures, S-nitrosylated HMGB1 induced stronger microglial activation and neurodegeneration than unmodified HMGB1. *In vivo*, intranigral injection of unmodified HMGB1 caused sustained microglial activation, dopaminergic neuronal loss, and motor impairment in wild-type mice but not in Mac1-deficient mice (35). These findings connect the modification state of HMGB1 with its secretion and Mac1-dependent activity.

Direct manipulation of extracellular HMGB1 has produced consistent effects in toxin-based PD models. Systemic HMGB1 neutralization reduced dopaminergic neuronal loss and RAGE/TNF-α-associated inflammatory signaling in MPTP-treated mice (60). In 6-hydroxydopamine-treated rats, an anti-HMGB1 monoclonal antibody additionally reduced microglial activation and blood–brain barrier disruption, preserved the nigrostriatal dopaminergic system, and improved behavioral performance (61). The HMGB1 A box, which competitively interferes with HMGB1 receptor engagement, also preserved nigrostriatal dopaminergic neurons in an MPTP model while reducing microglial cytokine production and T-cell infiltration (62). These intervention studies support an active contribution of extracellular HMGB1 to inflammatory neuronal injury.

### 4.2 α-Synuclein proteostasis, mitochondrial stress, and ferroptosis

HMGB1 interacts directly with α-synuclein and influences its autophagic clearance. HMGB1 was initially identified as a protein that preferentially binds filamentous α-synuclein over soluble monomers (63). In rotenone-treated dopaminergic cells, endogenous HMGB1 translocated to the cytoplasm, colocalized with α-synuclein, and competed with Vps34 for binding to Beclin1. The resulting disruption of the Beclin1-Vps34 complex impaired autophagic flux and increased α-synuclein accumulation (64). Paraquat similarly strengthened the interaction between HMGB1 and α-synuclein, disturbed HMGB1-Beclin1 complex formation, and reduced autophagic clearance in SH-SY5Y cells (65). SNCA overexpression was also associated with HMGB1-dependent autophagy inhibition, which was reversed by enhancing autophagic activity (66).

The direction of HMGB1-mediated autophagy regulation changes with the experimental context. Under starvation, increasing endogenous HMGB1 promoted the degradation of wild-type, A30P, and A53T α-synuclein through an Atg5-dependent pathway in SH-SY5Y cells. Atg5 knockdown abolished this effect (67). Thus, cytoplasmic HMGB1 accumulation during rotenone or paraquat exposure is associated with disruption of the Beclin1-Vps34 complex, whereas endogenous HMGB1 can support Atg5-dependent α-synuclein degradation during nutrient deprivation. The balance between these activities is determined by the cellular stressor, HMGB1 abundance and localization, and the availability of its binding partners.

Mitochondrial stress accompanies HMGB1-associated inflammation in PD models. In adult zebrafish exposed to MPTP, increased expression of hmgb1, tlr4, and nfkb occurred together with altered expression of the mitochondrial fission gene fis1 and the mitophagy-related genes pink1 and park2 (68). These coordinated transcriptional changes link the HMGB1 inflammatory response with mitochondrial fission and mitophagy-associated pathways during toxin-induced dopaminergic injury.

HMGB1 has also been associated with the ferroptosis-related transcriptional landscape of the human PD substantia nigra. Analysis of single-cell RNA-sequencing data identified changes in genes involved in iron handling, glutathione metabolism, and lipid peroxide control across dopaminergic neurons and glial populations. HMGB1 was increased across multiple cell types and was identified, together with ceruloplasmin and transferrin, as a secreted ferroptosis-related differentially expressed gene (69). This association places HMGB1 within the cellular response to iron-dependent oxidative stress in PD.

### 4.3 Disease-specific mechanistic integration

In PD, HMGB1 connects extracellular inflammatory propagation with intracellular proteostatic stress. Neuron- and glia-derived HMGB1 sustains Mac1-, RAGE-, and TLR4-associated signaling, and its neutralization or receptor antagonism reduces dopaminergic injury in toxin-based models (57–62). Within stressed cells, HMGB1 modifies α-synuclein autophagic processing in opposite directions according to the stimulus and autophagic state (63–67). Mitochondrial and ferroptosis-related transcriptomic changes extend this framework to oxidative stress (68, 69), while human biofluid and tissue studies associate total HMGB1 with clinical and pathological features of PD (59, 60, 70).

## 5 HMGB1 in amyotrophic lateral sclerosis

Amyotrophic lateral sclerosis (ALS) is characterized by the progressive degeneration of upper and lower motor neurons and by extensive interactions among motor neurons, astrocytes, microglia, and peripheral immune cells. Studies of human spinal cord tissue, cellular systems, and transgenic mice expressing human superoxide dismutase 1 in which glycine 93 is replaced by alanine [SOD1(G93A)] link HMGB1 to the propagation of neuronal stress and glial dysfunction through RAGE- and TLR4-associated signaling (10, 71). Across these settings, extracellular HMGB1 amplifies motor-neuron stress and glial dysfunction during disease progression.

### 5.1 Cellular sources and spatiotemporal redistribution

Postmortem spinal cord tissue from patients with ALS shows increased expression of HMGB1 and the receptor for advanced glycation end products (RAGE), together with other RAGE ligands such as S100 calcium-binding protein B (S100B) and carboxymethyllysine (72). In a separate analysis of spinal cord tissue from patients with sporadic ALS, Toll-like receptor 2 (TLR2), Toll-like receptor 4 (TLR4), RAGE, and HMGB1 were increased in reactive glial cells. TLR2 was detected predominantly in microglia and macrophages, whereas TLR4 and RAGE were prominent in astrocytes. Increased cytoplasmic HMGB1 immunoreactivity in reactive glia further indicated redistribution of the protein within the affected spinal cord (73).

HMGB1 localization also changes during disease progression in SOD1(G93A) mice. At the presymptomatic stage, HMGB1 distribution in the spinal cord is similar to that of control mice. During the symptomatic and terminal stages, HMGB1 immunoreactivity decreases in degenerating motor neurons and becomes prominent in reactive glial cells (74). A subsequent time-course analysis showed a progressive reduction in the proportion of motor neurons with nuclear HMGB1 and a corresponding increase in neurons with cytoplasmic HMGB1 from the asymptomatic stage to disease onset and the symptomatic stage (75). These findings place HMGB1 redistribution alongside the development of motor-neuron stress and glial activation.

HMGB1 abnormalities have also been identified in cell-specific models. N9 microglia expressing SOD1(G93A) show increased expression of HMGB1, S100B, inducible nitric oxide synthase, and RAGE, and their extracellular vesicles contain HMGB1 and mutant SOD1 (76). Cortical astrocytes isolated from SOD1(G93A) mice similarly exhibit increased HMGB1, S100B, connexin 43, and vimentin expression together with reduced microRNA-146a (miR-146a) (77). Restoring miR-146a normalizes astrocytic HMGB1 expression and reduces the adverse effects of the astrocyte secretome on motor-neuron-like cells and microglia (78). Motor neurons and glial cells therefore contribute distinct but connected components of HMGB1-associated signaling in ALS.

### 5.2 RAGE/TLR4 signaling and glial-neuronal interactions

The increased expression of RAGE, TLR4, and their ligands in ALS spinal cord has prompted direct examination of these receptors in SOD1(G93A) mice. RAGE deletion reduces spinal cord microgliosis and inflammatory gene expression, slows motor decline, and extends survival in this model (79). TLR4 deficiency similarly improves hindlimb grip strength and produces a modest extension of survival (80). These interventions identify RAGE- and TLR4-associated innate immune signaling as modifiers of disease progression.

Astrocytic responses to HMGB1 depend on cellular state. In normal spinal cord astrocytes, stimulation with dsHMGB1 increases the expression of brain-derived neurotrophic factor and glial cell line-derived neurotrophic factor. This neurotrophic response is markedly impaired in SOD1(G93A) astrocytes (75). HMGB1 signaling in ALS therefore involves both the accumulation of inflammatory signals and the loss of an astrocytic support response. The combination of neuronal HMGB1 redistribution, altered astrocytic responsiveness, and increased HMGB1-related signaling in microglia provides a route through which local motor-neuron stress can be transmitted across the spinal cord cellular environment.

Microglial and astrocytic HMGB1 abnormalities converge on non-cell-autonomous motor-neuron injury. SOD1(G93A) microglia display increased HMGB1 and RAGE expression and release extracellular vesicles containing inflammatory mediators (76), whereas the secretome of SOD1(G93A) astrocytes disrupts motor-neuron and microglial homeostasis (77, 78). Within this interaction, HMGB1 functions as one component of a broader network of extracellular signals that modifies glial reactivity and motor-neuron function.

### 5.3 Disease-stage effects, biomarker findings, and therapeutic response

Within the RAGE- and TLR4-associated receptor systems, HMGB1 acts alongside other endogenous ligands, including S100 proteins and advanced glycation end products. Deletion of RAGE or TLR4 reduces inflammatory signaling and extends survival in SOD1(G93A) mice, identifying these receptor pathways as modifiers of ALS progression (72, 73, 79, 80).

The most direct test of extracellular HMGB1 used the neutralizing antibody 2G7 in SOD1(G93A) mice. Treatment beginning at 35 days of age transiently preserved hindlimb grip strength at 56 and 63 days but did not improve overall motor scores, prevent weight loss, or extend survival. Treatment initiated at 70 days, when motor deficits were established, did not improve motor performance or survival. HMGB1 neutralization reduced spinal cord expression of tumor necrosis factor-α and complement C5a receptor 1 without producing a broad reduction in microglial or astrocytic activation (81). The treatment profile identifies extracellular HMGB1 as an early contributor to motor dysfunction, with little influence on survival-determining processes in SOD1(G93A) mice.

A study of 61 patients with ALS reported higher serum anti-HMGB1 autoantibody levels than in patients with Alzheimer’s disease, patients with Parkinson’s disease, and healthy controls. Autoantibody levels were elevated at early clinical stages and correlated with disease severity (82). This marker captures an immune response to HMGB1 and is distinct from circulating HMGB1 concentration or bioactivity.

Together, these findings position extracellular HMGB1 as a progression-associated inflammatory amplifier of glial-neuronal dysfunction in ALS. HMGB1 redistribution accompanies motor-neuron stress, and its neutralization transiently improves early motor performance without altering survival.

## 6 HMGB1 in Huntington’s disease and selected neurodegenerative disorders

HMGB1 has been examined in Huntington’s disease (HD), spinocerebellar ataxias (SCAs), and tau-associated frontotemporal dementia (FTD). In polyglutamine disease models, reduced soluble nuclear HMGB1 and disruption of its DNA- and protein-homeostasis functions have been described. In tauopathy models, extracellular HMGB1 participates in astrocyte senescence and paracrine inflammatory signaling.

### 6.1 Huntington’s disease: reduced soluble nuclear HMGB1 and proteostasis

HD is caused by CAG repeat expansion in the HTT gene, which produces huntingtin with an expanded polyglutamine (polyQ) tract (83). In a soluble nuclear proteomic analysis of primary neurons expressing mutant huntingtin or mutant ataxin-1, Qi et al. identified reductions in soluble nuclear HMGB1 and HMGB2. Immunoprecipitation and pulldown assays showed that HMGB proteins interacted with mutant huntingtin and ataxin-1, while immunohistochemistry demonstrated reduced HMGB1/2 outside inclusion bodies in affected neurons. Compensatory expression of HMGB proteins reduced polyQ-associated genotoxic stress and neuronal injury in primary-neuron and Drosophila models (84). In this experimental system, the reduction in soluble nuclear HMGB1/2 preceded microscopically visible inclusion formation.

HMGB1 also has chaperone-like activity relevant to polyQ proteostasis. HMGB1 directly interacts with expanded polyQ and inhibits polyQ-associated aggregation and cytotoxicity. HMGB1 overexpression or exposure to recombinant HMGB1 reduced aggregate formation in SH-SY5Y cells, HMGB1-deficient fibroblasts, and primary striatal neurons (85). A separate study identified HMGB1 as an interaction partner of the N17 and proline-tyrosine nuclear localization signal regions of huntingtin. This interaction was detected with both normal and mutant huntingtin, was enhanced by reactive oxygen species, and contributed to stress-induced huntingtin nuclear entry (86). HMGB1 therefore participates in both polyQ protein handling and the oxidative-stress response that regulates huntingtin localization.

Extracellular HMGB1-related signaling has been examined in 3-nitropropionic acid (3-NP)-induced striatal injury. In rats, 3-NP increased striatal HMGB1, TLR4, NF-κB, oxidative-stress, and cell-death-associated markers. Artemisinin treatment reduced HMGB1/TLR4/NF-κB and RIPK1/RIPK3 signaling together with improvements in motor and cognitive measures (87). Glycyrrhizin produced similar reductions in HMGB1/TLR4/NF-κB signaling, oxidative stress, inflammation, and apoptosis in a separate 3-NP model (88). These studies place HMGB1-associated inflammatory signaling within toxin-induced striatal degeneration.

### 6.2 Spinocerebellar ataxias: nuclear and mitochondrial HMGB1

Spinocerebellar ataxia type 1 (SCA1) provides an *in vivo* model of HMGB1 functional loss. Mutant ataxin-1 interacts with HMGB1 and impairs its contribution to mitochondrial DNA maintenance. In mutant ataxin-1 knock-in mice, reduced HMGB1 activity was associated with mitochondrial DNA damage and impaired mitochondrial function in cerebellar neurons. Cerebellar delivery of HMGB1 using an adeno-associated viral vector enhanced mitochondrial DNA repair, improved neuropathology and motor performance, and extended survival (89). This experiment provides a proof-of-concept for HMGB1 supplementation in a disease model characterized by reduced intracellular HMGB1 function.

A related mechanism has been described in spinocerebellar ataxia type 17 (SCA17), which is caused by polyQ expansion in the TATA-binding protein (TBP). In cellular models expressing polyQ-expanded TBP, mutant TBP aggregates incorporated HMGB1 and reduced the soluble HMGB1 pool. HMGB1 overexpression produced a modest reduction in mutant TBP aggregation and restored transcription from the heat shock protein family A member 5 (HSPA5) promoter. Reduced cytoplasmic HMGB1 was also associated with impaired autophagic responses during nutrient stress (90). Thus, HMGB1 abnormalities in SCA17 involve sequestration, transcriptional regulation, proteostasis, and stress-responsive autophagy.

The SCA1 and SCA17 studies describe distinct consequences of intracellular HMGB1 disruption. In SCA1 mice, HMGB1 supplementation improved mitochondrial DNA repair and disease-associated phenotypes *in vivo*. In SCA17 cells, HMGB1 sequestration altered HSPA5 transcription and polyQ protein handling. These observations support an intracellular protective role for HMGB1 in defined polyQ disease models.

### 6.3 Frontotemporal dementia and other tauopathies

Tau oligomers link HMGB1 release to astrocyte senescence in tau-associated FTD. Postmortem brain tissue from patients with FTD or Alzheimer’s disease contains tau oligomer-associated astrocytes expressing markers of cell-cycle arrest, DNA damage, and the senescence-associated secretory phenotype (SASP), including HMGB1. Direct exposure to tau oligomers induces senescence and HMGB1 release in cultured astrocytes, and the released HMGB1 promotes a senescence-like phenotype in neighboring astrocytes (49).

Inhibition of HMGB1 release with ethyl pyruvate and glycyrrhizic acid reduced p38 mitogen-activated protein kinase and NF-κB signaling, SASP-associated molecules, and paracrine senescence in cultured cells. In hTau mice, the same treatment reduced astrogliosis, neuroinflammation, tau oligomers, hyperphosphorylated tau, and neurofibrillary tangle burden, with concurrent improvement in learning and memory (49). HMGB1 release therefore participates in a tau oligomer-astrocyte senescence pathway that links local tau pathology to inflammatory signaling in tau-associated FTD.

Across the disorders discussed in this section, intracellular HMGB1 participates in polyQ protein handling, DNA and mitochondrial DNA repair, and transcriptional regulation. Filamentous α-synuclein also binds HMGB1 preferentially over monomeric α-synuclein, complementing the stress-dependent HMGB1-α-synuclein interactions discussed in Section 4 (63–67). Extracellular HMGB1 contributes to inflammatory signaling in 3-NP-induced striatal injury and to tau oligomer-induced astrocyte senescence. The AAV-HMGB1 experiment in SCA1 provides an *in vivo* proof-of-concept for restoring intracellular HMGB1 function, whereas the tauopathy studies define a distinct role for HMGB1 release in intercellular stress signaling.

## 7 Cross-disease integration of HMGB1 pathobiology

Across neurodegenerative disorders, HMGB1 participates in two recurrent compartment-associated processes: disruption of its intracellular homeostatic functions and activation of extracellular inflammatory signaling. The relative contribution of each process varies with the disease mechanism, cellular source, and stage of neuronal injury. Reduced soluble nuclear HMGB1 is most clearly represented in polyglutamine disease models, whereas HMGB1 redistribution and extracellular signaling have been characterized more extensively in AD, PD, ALS, and tau-associated FTD. These processes can occur within the same disorder as cellular stress redistributes HMGB1 between the nucleus, cytoplasm, and extracellular space.

### 7.1 Compartment-dependent patterns across neurodegenerative diseases

Polyglutamine models provide the clearest examples of impaired intracellular HMGB1 function. Mutant huntingtin, ataxin-1, and polyQ-expanded TATA-binding protein reduce or sequester soluble HMGB1 and disrupt its chaperone, genome-maintenance, mitochondrial, transcriptional, and autophagic functions. HMGB1 restoration improves cellular phenotypes and, in SCA1 mice, neurological outcomes and survival (84, 85, 89, 90).

In AD, PD, ALS, and tau-associated FTD, HMGB1 redistribution and release are more prominent. Extracellular HMGB1 modifies Aβ clearance, glial activation, neuronal DNA repair, and tau-associated astrocyte senescence in AD and FTD (45–55); it sustains microglial inflammatory signaling and participates in stress-dependent α-synuclein processing in PD (57–67); and its neuronal-to-glial redistribution accompanies disease progression in ALS (72–78). Disruption of intracellular HMGB1 and extracellular inflammatory signaling can therefore coexist within the same disease.

### 7.2 Cellular sources and intercellular propagation

The cellular origin of HMGB1 shapes how local injury is transmitted through neural tissue. Neuronal stress and lytic death release intracellular HMGB1 into the extracellular environment, whereas activated microglia and astrocytes can mobilize and secrete HMGB1 through regulated pathways. Once released, HMGB1 acts on neurons, glia, endothelial cells, and infiltrating immune cells according to the receptor repertoire of each target population.

In AD and tau-associated FTD, astrocytic or neuronal HMGB1 released in response to tau oligomers, APOE4, or neuronal stress extends senescence, gliosis, neurite injury, and impaired DNA repair to neighboring cells (49, 50, 52–55). In PD, HMGB1 released from injured neurons and activated microglia sustains Mac1-, RAGE-, and TLR4-associated microglial activation (57–59). In ALS, loss of motor-neuron nuclear HMGB1 coincides with cytoplasmic redistribution and altered astrocytic and microglial signaling (74–78).

Intervention studies further distinguish HMGB1-specific signaling from broader receptor activity. HMGB1 neutralization reduces dopaminergic injury in PD models and transiently improves early motor function in SOD1(G93A) mice, whereas RAGE or TLR4 deletion has broader effects on ALS progression because both receptors integrate additional endogenous ligands (60–62, 79–81). Across diseases, HMGB1 propagation depends on the source cell, mode of release, and receptor repertoire of the target cell.

### 7.3 Neurovascular and peripheral-central signaling

The neurovascular interface extends HMGB1 signaling beyond local neuron-glia interactions. In human brain microvascular endothelial monolayers, HMGB1 and thrombin increase paracellular permeability and disrupt the distribution of the tight-junction protein zonula occludens-1 (15). Endothelial barrier dysfunction can thereby alter communication between circulating inflammatory mediators and the central nervous system, while HMGB1 released within the brain can act directly on vascular cells. This positions the blood-brain barrier as both a target of extracellular HMGB1 and an interface through which local and systemic inflammatory responses converge.

Peripheral HMGB1-associated communication also involves the intestine and spleen. In mice with chronic colitis, serum and hippocampal HMGB1 increased together with blood-brain barrier permeability, hippocampal caspase-1/caspase-11/gasdermin activation, and memory impairment, linking chronic intestinal inflammation with systemic and central HMGB1-associated responses (91).

The spleen contributes to peripheral-central regulation through control of circulating protein and immune signals. In APP/PS1 mice, splenic monocytes and macrophages cleared circulating Aβ, whereas splenectomy increased blood and brain Aβ and aggravated cognitive and neuropathological changes (92). In a separate model of persistent neuroinflammation, circulating HMGB1 and splenic Hmgb1 transcription increased after peripheral immune stimulation, and intravenously administered HMGB1 induced inflammatory gene expression in the hippocampus, cortex, and midbrain (93). Taken together, these findings identify splenic immune regulation and circulating HMGB1 as complementary components of peripheral-central inflammatory communication.

The lung-brain axis provides a disease-specific example of peripheral HMGB1-associated signaling. In 5xFAD mice, ozone exposure increases amyloid pathology and alters plaque-associated astrocyte and microglial responses (56, 94). Deletion of Hmgb1 from peripheral myeloid cells modifies the induction of disease-associated astrocyte markers after ozone exposure, connecting a peripheral myeloid HMGB1 response with the cerebral plaque microenvironment (56). Peripheral HMGB1 signaling can therefore influence central glial states without requiring its production by neurons or resident glia. Together with endothelial barrier responses, this pathway broadens HMGB1-associated pathology from local intercellular signaling to communication between peripheral immune compartments and the brain.

The central and peripheral HMGB1-associated signaling routes are summarized in **Figure 2**.

**Figure 2:**
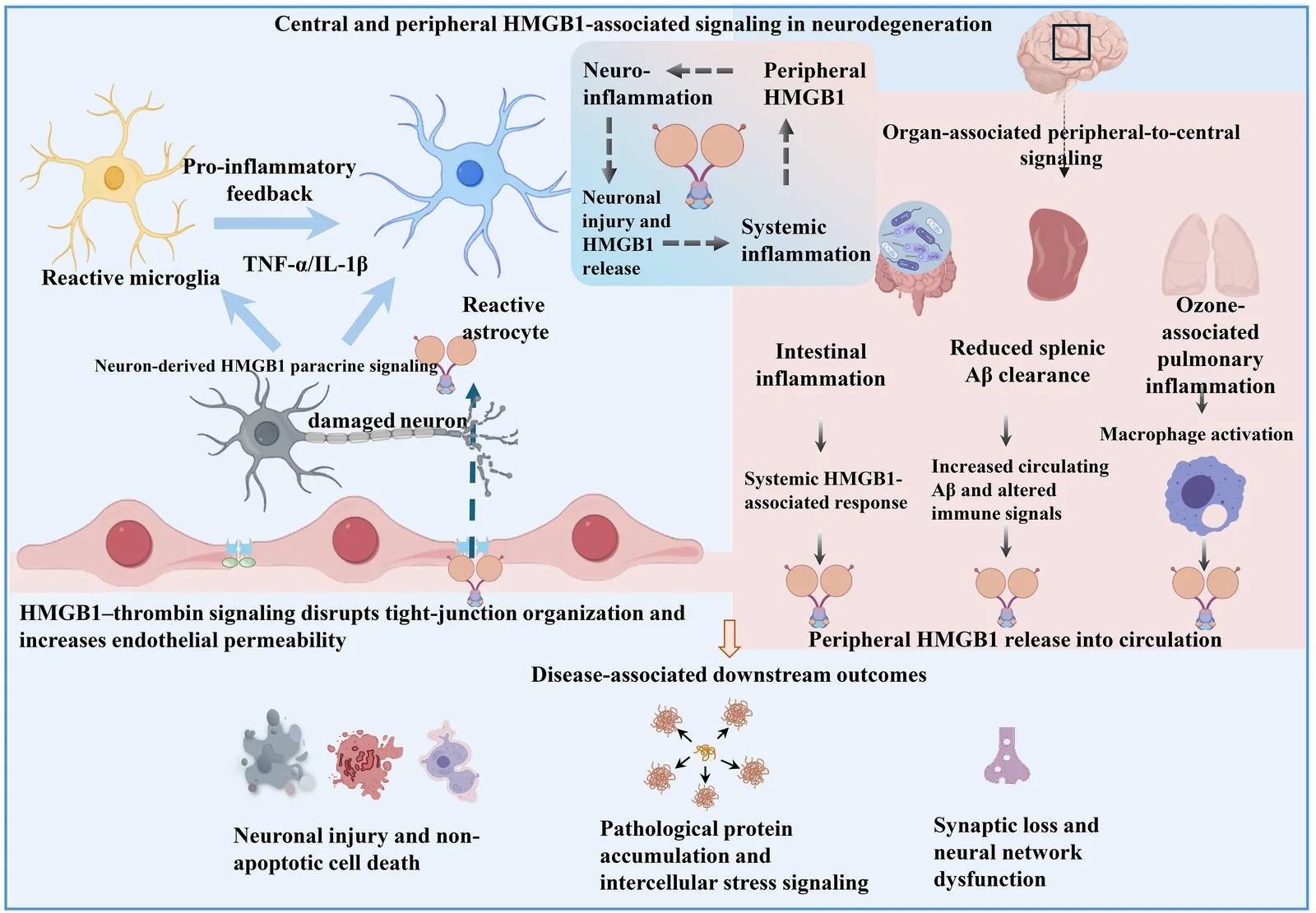
Central and peripheral HMGB1-associated signaling in neurodegeneration. Within the central nervous system, HMGB1 released from injured neurons and activated glial cells participates in paracrine and autocrine signaling among neurons, microglia, and astrocytes, sustaining glial reactivity and cytokine production. HMGB1 and thrombin disrupt endothelial tight-junction organization and increase blood–brain barrier permeability. Intestinal inflammation is associated with increased circulating and hippocampal HMGB1, whereas ozone-associated pulmonary inflammation engages peripheral myeloid HMGB1 signaling. Reduced splenic Aβ clearance increases circulating Aβ and alters peripheral immune signals. These central and peripheral processes influence neuronal injury and non-apoptotic cell death, pathological protein accumulation and intercellular stress signaling, and synaptic and neural-network dysfunction. Solid arrows indicate local cellular or molecular signaling, whereas dashed arrows indicate communication between central and peripheral inflammatory compartments. Aβ, amyloid-β; HMGB1, high-mobility group box 1; IL-1β, interleukin-1β; TNF-α, tumor necrosis factor-α.

### 7.4 Disease stage and the causal position of HMGB1

The position of HMGB1 within the pathological sequence differs across diseases. In polyQ models, reduced soluble nuclear HMGB1 and disruption of its intracellular functions occur alongside early genotoxic and proteostatic stress (84). Restoration of HMGB1 improves mitochondrial DNA repair and disease-associated phenotypes in SCA1 mice (89), while HMGB1 sequestration alters transcription and protein handling in SCA17 cells (90). In these systems, HMGB1 functions as an intracellular maintenance factor whose depletion contributes directly to neuronal vulnerability.

In AD, HMGB1 acts at several points in the disease process. Extracellular HMGB1 stabilizes Aβ assemblies and impairs microglial Aβ clearance (45–47), tau oligomers and APOE4 promote its release from astrocytes or neurons (49, 50), and neuron-derived HMGB1 participates in TLR4-dependent neurite injury and DNA repair impairment within the phase 0 and TRIAD models (52–55). In PD, extracellular HMGB1 propagates inflammatory signals after dopaminergic neuronal injury, whereas cytoplasmic HMGB1 modifies α-synuclein processing according to the cellular stressor and autophagic state (57, 58, 64–67). These diseases therefore combine stress-dependent HMGB1 redistribution with distinct intracellular and extracellular effects.

In ALS, HMGB1 redistribution accompanies motor-neuron stress, and extracellular HMGB1 contributes to an early component of motor dysfunction. The transient motor benefit and unchanged survival after 2G7 treatment identify HMGB1 as a progression-associated inflammatory amplifier within the SOD1(G93A) model (81). In tau-associated FTD, tau oligomer-induced HMGB1 release acts downstream of protein stress and sustains astrocyte senescence and paracrine inflammatory signaling (49). Across these disorders, HMGB1 can occupy three positions in the pathological sequence: an intracellular maintenance factor, a mediator released during cellular stress or injury, and an extracellular amplifier of multicellular dysfunction. The relative contribution of these positions is determined by disease mechanism, cellular source, and stage of tissue injury.

A compartment- and stage-resolved interpretation reconciles the protective intracellular functions of HMGB1 with its extracellular inflammatory activity. It also provides the biological basis for separating approaches that restore intracellular HMGB1 function from those that selectively inhibit extracellular signaling.

## 8 HMGB1 biomarkers and therapeutic translation

Clinical translation of HMGB1 biology requires separation of three related but distinct questions: whether HMGB1 reflects tissue injury or inflammatory activity, whether a measurement identifies a functionally defined HMGB1 state, and whether an intervention acts on intracellular or extracellular HMGB1. Current clinical studies have mainly addressed the first question, whereas most therapeutic studies remain preclinical and have not resolved the biochemical form or cellular source of the targeted HMGB1 pool.

### 8.1 HMGB1 biomarkers and redox isoform detection

Human studies have examined HMGB1 in blood, cerebrospinal fluid, and postmortem tissue, most often using immunoassays that quantify total HMGB1. In mild cognitive impairment (MCI), an interaction between apolipoprotein E4 (APOE4) and plasma HMGB1 was associated with widespread cortical thinning (51). In Parkinson’s disease (PD), a study of 75 patients and 47 controls detected higher total HMGB1 in both serum and cerebrospinal fluid. Serum HMGB1 correlated with disease duration, but neither serum nor cerebrospinal fluid HMGB1 correlated with Hoehn and Yahr stage (60). A separate study of 120 patients with PD and 100 controls reported higher serum HMGB1 and Toll-like receptor 4 (TLR4), with both measures increasing across clinical stages and disease duration (59). Serum HMGB1 has also been associated with retinal ganglion cell-inner plexiform layer thinning and cognitive impairment in PD (70), while a smaller case-control study found higher serum HMGB1 and high-sensitivity C-reactive protein in patients with PD than in matched controls (95).

A study of 61 patients with amyotrophic lateral sclerosis (ALS) reported higher serum anti-HMGB1 autoantibody levels than in patients with Alzheimer’s disease, patients with PD, and healthy controls. Autoantibody levels were elevated at early clinical stages and correlated with disease severity (82). This marker captures an immune response to HMGB1 and is distinct from circulating HMGB1 concentration or bioactivity. Representative human findings are summarized in **Table 2**.

**Table 2:** HMGB1-related measurements reported in human neurodegenerative disease studies.

Disease or clinical context	Specimen and analyte	Study population	Principal finding	Redox isoform resolved	Ref.
Mild cognitive impairment	Plasma total HMGB1 and APOE4 genotype	27 participants with MCI and 25 cognitively normal controls	Within the MCI group, the interaction between APOE4 and plasma HMGB1 was associated with extensive cortical thinning involving frontal, temporal, and insular regions	No	(51)
Parkinson’s disease	Serum and cerebrospinal fluid total HMGB1; postmortem substantia nigra HMGB1	75 patients with PD and 47 controls for biofluid analysis; six PD and five control substantia nigra samples	HMGB1 was increased in serum, cerebrospinal fluid, and substantia nigra; serum HMGB1 correlated with disease duration, but neither serum nor cerebrospinal fluid HMGB1 correlated with Hoehn and Yahr stage	No	(60)
Parkinson’s disease	Serum total HMGB1 and TLR4	120 patients with PD and 100 healthy controls	HMGB1 and TLR4 were higher in PD and increased with clinical stage and disease duration	No	(59)
Parkinson’s disease with cognitive impairment	Serum total HMGB1 and retinal GCIPL thickness	47 patients with PD and 42 healthy controls	Higher serum HMGB1 was associated with disease duration, motor impairment, lower cognitive scores, and retinal thinning	No	(70)
Parkinson’s disease	Serum total HMGB1 and high-sensitivity C-reactive protein	30 patients with PD and 30 matched controls	Both markers were higher in PD; serum HMGB1 showed modest diagnostic discrimination in this cohort	No	(95)
Amyotrophic lateral sclerosis	Serum anti-HMGB1 autoantibody	61 patients with ALS; 40 patients with Alzheimer’s disease, 40 patients with PD, and 40 healthy controls	Autoantibody levels were increased at early clinical stages and correlated with greater disease severity	Not applicable: the assay measured autoantibody rather than an HMGB1 isoform	(82)

ALS, amyotrophic lateral sclerosis; APOE4, apolipoprotein E4; GCIPL, ganglion cell-inner plexiform layer; HMGB1, high-mobility group box 1; MCI, mild cognitive impairment; PD, Parkinson’s disease; TLR4, Toll-like receptor 4.

Total HMGB1 is not specific to neurodegeneration. Its circulating concentration increases in acute tissue injury, infection, autoimmune inflammation, and systemic inflammatory states, and peripheral sources can contribute substantially to blood measurements (7, 42). A single total-HMGB1 value therefore does not distinguish central from peripheral release, active secretion from passive release, or reparative from cytokine-inducing extracellular activity.

These distinctions depend partly on the redox state of Cys23, Cys45, and Cys106. Fully reduced HMGB1 (frHMGB1), disulfide HMGB1 (dsHMGB1), and terminally oxidized HMGB1 (oxHMGB1) differ in receptor engagement and extracellular function (28–32). Conventional enzyme-linked immunosorbent assays and immunoblots detect immunoreactive HMGB1 but do not determine the oxidation state of individual cysteine residues. Redox-resolved analysis requires rapid stabilization of thiols during sample collection, HMGB1 enrichment, differential cysteine alkylation, proteolytic digestion, and liquid chromatography-tandem mass spectrometry of cysteine-containing peptides (29).

Clinical implementation is limited by the low abundance of individual isoforms, oxidation or reduction during sample handling, variable immunoenrichment recovery, and the absence of shared calibrators and interlaboratory quality-control standards. Sample matrix, processing interval, storage conditions, cellular contamination, and repeated freeze-thaw cycles can alter the measured isoform distribution. None of the neurodegenerative disease cohorts summarized in **Table 2** quantified frHMGB1, dsHMGB1, and oxHMGB1 separately. Consequently, the dsHMGB1/frHMGB1 ratio cannot currently be used as a validated clinical measure of disease stage.

### 8.2 HMGB1-targeted interventions: preclinical and clinical findings

HMGB1-directed interventions fall into three principal categories: neutralization of extracellular HMGB1, modulation of HMGB1 release or receptor signaling, and restoration of intracellular HMGB1 function. These approaches act on different molecular pools and cannot be considered pharmacologically equivalent.

In 5xFAD mice, subcutaneous administration of an anti-HMGB1 monoclonal antibody reduced TLR4-myristoylated alanine-rich C-kinase substrate (MARCKS)-associated neurite degeneration and improved dendritic-spine density and cognitive performance (53). In an AlCl_3_/D-galactose rat model, weekly intraperitoneal administration of calcium phosphate nanoparticle-encapsulated HMGB1 Box A plasmids improved behavioral performance and reduced amyloid and tau pathology, glial activation, cellular senescence, and oxidative DNA damage (96). Direct engagement of extracellular HMGB1 was not measured in the latter study.

In toxin-based PD models, anti-HMGB1 monoclonal antibody, glycyrrhizin, and recombinant HMGB1 Box A protein reduced glial activation and dopaminergic neuronal injury (60–62, 97). In SOD1(G93A) mice, the 2G7 antibody transiently improved early motor performance without extending survival (81). The treatment profile identifies extracellular HMGB1 as an early contributor to motor dysfunction, with little influence on survival-determining processes in SOD1(G93A) mice.

Glycyrrhizin binds HMGB1 but also affects oxidative, metabolic, and inflammatory pathways. Its effects in MPTP-based PD models and 3-nitropropionic acid-induced striatal injury therefore demonstrate pharmacological modulation of an HMGB1-associated response rather than selective inhibition of a single HMGB1 isoform (60, 88, 97). RAGE or TLR4 deletion extends survival in SOD1(G93A) mice (79, 80), but both receptors recognize ligands other than HMGB1. Receptor deletion consequently defines the contribution of broader inflammatory signaling systems rather than HMGB1-specific target engagement.

Restoration of intracellular HMGB1 has been examined most directly in spinocerebellar ataxia type 1. Adeno-associated viral delivery of full-length HMGB1 improved mitochondrial DNA repair, motor performance, cerebellar pathology, and survival in mutant ataxin-1 knock-in mice (89). This intervention addresses depletion of intracellular HMGB1 function and is mechanistically distinct from extracellular neutralization or administration of an HMGB1 Box A antagonist.

Cell-specific genetic studies provide additional information about HMGB1 source. Microglia-specific Hmgb1 deletion delayed symptom onset, reduced demyelination, and altered microglial and macrophage inflammatory responses in experimental autoimmune encephalomyelitis (98). In cerebral ischemia-reperfusion, microglia-specific Hmgb1 deletion reduced early lesion volume, acute neurological deficits, microglial inflammatory activation, and neuronal injury (99). These studies identify microglial HMGB1 as an active component of inflammatory amplification in demyelinating and acute ischemic injury models.

Human intervention data are limited to drugs with multiple molecular actions. In a randomized study of 62 patients with PD, adjunctive pentoxifylline reduced serum HMGB1 and improved Unified Parkinson’s Disease Rating Scale scores over six months (100). In a randomized pilot study, adjunctive metformin reduced serum HMGB1, TLR4, and α-synuclein over three months, although the change in Unified Parkinson’s Disease Rating Scale scores did not differ significantly between treatment groups (101). In both studies, circulating HMGB1 was a secondary pharmacodynamic measure. Neither drug selectively neutralizes extracellular HMGB1, and neither study determined HMGB1 redox state or central target engagement. Representative interventions are summarized in **Table 3**.

**Table 3:** Representative HMGB1-associated interventions in neurodegenerative and related neurological models.

Intervention	Disease or model	HMGB1-related intervention or pathway	Main outcome	Study context	Ref.
Anti-HMGB1 monoclonal antibody	5xFAD mouse model of AD	Extracellular HMGB1 and TLR4-MAPK-MARCKS signaling; HMGB1-Aβ interactions were also examined	Reduced MARCKS phosphorylation and neurite degeneration and improved dendritic-spine density and cognitive performance	Preclinical	(53)
Calcium phosphate nanoparticle-encapsulated HMGB1 Box A plasmid	AlCl_3_/D-galactose rat model	HMGB1 Box A expression; extracellular HMGB1 antagonism was proposed, but direct target engagement was not measured	Improved behavioral performance and reduced amyloid and tau pathology, glial activation, cellular senescence, and oxidative DNA damage	Preclinical	(96)
Anti-HMGB1 monoclonal antibody	6-hydroxydopamine rat model of PD	Extracellular HMGB1-associated inflammatory signaling	Preserved nigrostriatal dopaminergic neurons and terminals and reduced microglial activation, inflammatory cytokines, behavioral impairment, and blood-brain barrier disruption	Preclinical	(61)
Glycyrrhizin	MPTP mouse and zebrafish PD models	HMGB1-associated inflammatory signaling; glycyrrhizin also acts on additional molecular pathways	Preserved dopaminergic neurons and reduced inflammatory, apoptotic, vascular, and locomotor abnormalities	Preclinical	(60, 97)
Recombinant HMGB1 Box A protein	MPTP mouse model	Competitive inhibition of HMGB1 receptor engagement	Reduced microglial activation and T-cell infiltration and restored dopaminergic neurons and striatal fibers	Preclinical	(62)
2G7 neutralizing antibody	SOD1(G93A) ALS model	Extracellular HMGB1	Transient improvement in early motor performance without prolonged survival	Preclinical	(81)
RAGE or TLR4 deletion	SOD1(G93A) ALS model	HMGB1-associated, multi-ligand receptor signaling	Reduced inflammatory activity and extended survival	Genetic intervention; not HMGB1-specific	(79, 80)
AAV-mediated full-length HMGB1 delivery	Mutant ataxin-1 knock-in SCA1 model	Restoration of intracellular HMGB1 function	Improved mitochondrial DNA repair, motor function, cerebellar pathology, and survival	Preclinical	(89)
Microglia-specific Hmgb1 deletion	Experimental autoimmune encephalomyelitis	Microglial HMGB1	Delayed symptom onset and reduced inflammatory activity and demyelination	Cell-specific genetic intervention	(98)
Microglia-specific Hmgb1 deletion	Cerebral ischemia-reperfusion	Microglial HMGB1	Reduced hyperacute inflammation, neuronal injury, lesion volume, and neurological deficits	Cell-specific genetic intervention	(99)
Adjunctive pentoxifylline	Parkinson’s disease	Multiple inflammatory and metabolic pathways; serum HMGB1 measured as a pharmacodynamic variable	Reduced serum HMGB1 and improved clinical scores	Human randomized study	(100)
Adjunctive metformin	Parkinson’s disease	Multiple metabolic and inflammatory pathways; serum HMGB1 measured as a pharmacodynamic variable	Reduced serum HMGB1 without a significant between-group difference in clinical scores	Human randomized pilot study	(101)

AAV, adeno-associated virus; AD, Alzheimer’s disease; ALS, amyotrophic lateral sclerosis; HMGB1, high-mobility group box 1; MAPK, mitogen-activated protein kinase; MARCKS, myristoylated alanine-rich C-kinase substrate; MPTP, 1-methyl-4-phenyl-1,2,3,6-tetrahydropyridine; PD, Parkinson’s disease; RAGE, receptor for advanced glycation end products; SCA1, spinocerebellar ataxia type 1; TLR4, Toll-like receptor 4.

### 8.3 Safety, blood-brain barrier delivery, and therapeutic windows

The safety profile of an HMGB1-directed intervention depends on the molecular pool it reaches. Nuclear HMGB1 contributes to chromatin organization and DNA repair, whereas extracellular HMGB1 encompasses redox forms with inflammatory, chemotactic, or inactive properties (7, 28–30, 36, 39). Complete genetic depletion of HMGB1 is therefore not pharmacologically equivalent to extracellular neutralization. Conventional antibodies are not expected to enter intact cells efficiently, and their effects do not imply direct inhibition of nuclear HMGB1. Small molecules such as glycyrrhizin have broader intracellular and extracellular actions and require separate assessment of target selectivity.

Extracellular HMGB1 also participates in cell recruitment through the frHMGB1-CXCL12-CXCR4 complex (28–30). An antibody that recognizes multiple extracellular HMGB1 forms could inhibit both dsHMGB1-associated cytokine signaling and frHMGB1-associated chemotaxis. The long-term effects of sustained extracellular HMGB1 neutralization on host defense, tissue repair, and neural stress responses have not been characterized in the neurodegenerative disease studies summarized here. Existing intervention studies primarily report short-term neurological, histological, or inflammatory outcomes (17, 53, 60–62, 81, 97).

Blood-brain barrier permeability presents a separate constraint. Large antibodies have limited passive entry into the intact central nervous system. Barrier dysfunction occurs in neurodegenerative disease but varies among brain regions and across the disease course (15). The effects of systemically administered antibodies in toxin-based models may involve entry at sites of barrier disruption, neutralization of HMGB1 at the neurovascular interface, or modulation of peripheral inflammatory signaling. Demonstration of central target engagement requires measurement of antibody concentrations in cerebrospinal fluid or brain tissue, parenchymal distribution, receptor occupancy, and suppression of an HMGB1-dependent downstream response.

Small molecules may achieve greater central exposure, but blood-brain barrier penetration does not confer HMGB1 selectivity. Likewise, receptor-mediated transport, intranasal administration, and nanoparticle delivery remain delivery platforms rather than established solutions. Their evaluation requires pharmacokinetic comparison of blood, cerebrospinal fluid, and brain exposure together with assessment of intracellular and extracellular HMGB1 functions.

The intervention window also depends on disease mechanism. Restoration of intracellular HMGB1 is supported by the mutant ataxin-1 model, whereas extracellular neutralization has been examined mainly after inflammatory signaling or neuronal injury is established. A transition point between these strategies has not been defined in human neurodegenerative disease. Total circulating HMGB1 cannot identify that transition because it does not specify cellular source, subcellular redistribution, or redox state.

### 8.4 Disease staging and biomarker-guided trial design

Clinical disease stage and HMGB1 biochemical state are not interchangeable. Established clinical scales, neuroimaging, and disease-specific protein biomarkers describe symptom burden or pathological progression. An HMGB1-defined stage would require a reproducible relationship among subcellular redistribution, extracellular concentration, redox isoform distribution, and subsequent clinical change.

Current clinical findings do not establish such a relationship. Serum HMGB1 increased with PD stage in one cohort (59), whereas another study found no association between serum or cerebrospinal fluid HMGB1 and Hoehn and Yahr stage (60). Serum HMGB1 distinguished cognitive subgroups in a separate PD cohort, but the study was cross-sectional (70). Plasma HMGB1-APOE4 interaction in MCI and anti-HMGB1 autoantibody levels in ALS provide disease-associated measurements without defining a biochemical transition from intracellular HMGB1 loss to extracellular inflammatory activity (51, 82).

Biomarker-guided trials can use HMGB1 most directly as an exploratory marker of inflammatory activity or pharmacodynamic response. Disease-specific clinical and molecular criteria remain the basis for enrollment and staging. Serial sampling can determine whether changes in HMGB1 precede, accompany, or follow changes in established disease markers. For extracellular antagonists, informative pharmacodynamic measures include circulating drug concentration, cerebrospinal fluid exposure, total and redox-resolved HMGB1, and suppression of the receptor pathway targeted by the intervention. For intracellular restoration strategies, target engagement requires demonstration of cellular delivery, nuclear or mitochondrial localization, and recovery of the intended genome-maintenance or proteostatic function.

A clinically actionable HMGB1 assay would require standardized sample collection and processing, redox-state stabilization, isoform-specific quantification, reference materials, interlaboratory validation, and longitudinal association with disease-specific outcomes. Until these conditions are met, total HMGB1 is better suited to multimarker analyses than to independent diagnosis, disease staging, or selection between HMGB1-enhancing and HMGB1-inhibiting treatments.

### 8.5 Limitations of the current literature

The human literature is dominated by small or moderate-sized, single-center, cross-sectional studies. Most assays quantify total HMGB1 without resolving redox isoform, cellular source, mode of release, or HMGB1-containing molecular complexes. Reported associations with disease stage also differ among cohorts, and diagnostic thresholds have not undergone multicenter validation. The anti-HMGB1 autoantibody measured in ALS represents a host immune response and cannot be interpreted as a direct measure of extracellular HMGB1 concentration or activity.

Most intervention studies use transgenic or toxin-based models. SOD1(G93A) mice model a defined SOD1-associated form of ALS, while MPTP, 6-hydroxydopamine, paraquat, 3-nitropropionic acid, and AlCl_3_/D-galactose produce relatively rapid and experimentally controlled injury. These systems are informative for pathway analysis but do not reproduce the full temporal and molecular heterogeneity of sporadic human neurodegeneration. The phase 0 and transcriptional repression-induced atypical cell death models in AD and frontotemporal dementia have also been developed mainly within one research program (52–55). Experimental autoimmune encephalomyelitis and cerebral ischemia-reperfusion demonstrate cell-specific functions of microglial HMGB1 in demyelinating and acute injury settings, respectively; their temporal profiles differ from chronic neurodegenerative disease.

The relative contributions of central and peripheral HMGB1 have not been quantified across disease stages. Similarly, the effect of the gut microbiome on HMGB1 redox state has not been directly established in the neurodegenerative disorders covered here. Current findings support communication among intestinal, splenic, pulmonary, vascular, and central inflammatory compartments but do not assign a fixed proportion of disease progression to peripheral HMGB1.

## 9 Research priorities and conclusions

### 9.1 Priorities for mechanistic research

Mechanistic studies of HMGB1 require temporal and compartment-specific experimental designs. In AD models, extracellular HMGB1 participates in TLR4-MARCKS-associated neurite degeneration, while HMGB1-Ku70 signaling links neuronal HMGB1 release to impaired DNA repair (53, 55). In SOD1(G93A) mice, extracellular HMGB1 neutralization transiently improves early motor performance without extending survival (81). By contrast, adeno-associated viral delivery of HMGB1 in mutant ataxin-1 mice restores mitochondrial DNA repair and improves neurological and survival outcomes (89). These distinct treatment profiles support separate manipulation of nuclear, cytoplasmic, and extracellular HMGB1 at defined stages, accompanied by sequential measurements of HMGB1 redistribution, glial activation, neuronal injury, and functional decline. This approach can establish when HMGB1 participates in disease initiation and when it amplifies established tissue damage.

Cellular source and biochemical state require the same experimental resolution. Conditional deletion of microglial Hmgb1 alters inflammatory responses and neuronal injury in experimental autoimmune encephalomyelitis and cerebral ischemia-reperfusion (98, 99). Parallel manipulation of neuronal, microglial, astrocytic, and endothelial HMGB1 can be combined with measurements of subcellular localization, secretion, and receptor-pathway activation in chronic neurodegenerative models. Redox characterization at Cys23, Cys45, and Cys106 is essential because frHMGB1, dsHMGB1, and oxHMGB1 differ in receptor engagement and extracellular activity (28–32). Measurements of HMGB1-CXCL12 complexes and HMGB1 interactions with Aβ or α-synuclein can further distinguish receptor-active and protein-associated HMGB1 pools from total immunoreactive HMGB1 (30, 45–47, 63).

Cross-platform replication is a priority for mechanisms that organize disease-specific interpretations of HMGB1. The phase 0/TRIAD and HMGB1-Ku70 pathways described in AD models can be examined in independent human-derived neurons, glial co-cultures, organoids, and longitudinal animal studies (52, 54, 55). The reduction of soluble nuclear HMGB1 and its associated genome-maintenance and chaperone functions in polyglutamine and spinocerebellar ataxia models can similarly be evaluated across genotypes and disease stages (84–86, 89, 90). For central-peripheral signaling, source-tracing experiments and paired sampling of blood, cerebrospinal fluid, and brain tissue can quantify the contributions of circulating and central nervous system-derived HMGB1. Studies of chronic intestinal inflammation, splenic function, circulating HMGB1, and ozone-associated pulmonary injury provide experimental entry points for this analysis (56, 91–94). Incorporating redox-resolved measurements will determine whether intestinal or systemic inflammatory states alter HMGB1 biochemical activity before changes in central pathology.

### 9.2 Priorities for biomarker and therapeutic development

Human studies have associated circulating or cerebrospinal fluid HMGB1 and anti-HMGB1 autoantibodies with clinical features in mild cognitive impairment, PD, and ALS (51, 59, 60, 70, 82, 95). Biomarker development can now proceed through standardized sample collection, rapid thiol stabilization, reproducible HMGB1 enrichment, reference calibrators, and interlaboratory quality control. Redox-resolved liquid chromatography-tandem mass spectrometry provides a biochemical basis for distinguishing functional HMGB1 forms (29). Serial measurements in multicenter longitudinal cohorts, integrated with disease-specific protein biomarkers, imaging, and clinical outcomes, can establish whether HMGB1 contributes information about inflammatory activity, disease progression, or pharmacodynamic response.

Therapeutic development can be divided into extracellular antagonism and restoration of intracellular HMGB1 function. Antibodies and HMGB1 Box A have been examined as extracellular interventions in AD, PD, and ALS models (53, 61, 62, 81), whereas HMGB1 delivery in SCA1 addresses loss of intracellular genome-maintenance functions (89). For extracellular antagonists, informative endpoints include redox-form binding, pharmacokinetics, cerebrospinal fluid and brain exposure, suppression of the intended receptor pathway, and long-term effects on immune and tissue-repair responses. Intracellular restoration strategies require confirmation of cellular delivery, nuclear or mitochondrial localization, and recovery of the intended DNA-repair or proteostatic function. Cell-specific genetic studies can guide the selection of cellular targets and distinguish local target engagement from systemic anti-inflammatory effects (98, 99).

Pentoxifylline and metformin studies in PD demonstrate the feasibility of serial serum HMGB1 measurement as a pharmacodynamic endpoint in clinical research (100, 101). Trials of HMGB1-directed agents can extend this approach by measuring drug exposure, redox-resolved HMGB1, and activity of the receptor pathway targeted by the intervention. Patient enrollment and clinical staging can remain anchored to established disease-specific criteria, with HMGB1 incorporated as an exploratory stratification and pharmacodynamic variable. Longitudinal relationships among HMGB1 biochemical state, target engagement, and clinical change can then define the settings in which HMGB1-guided treatment selection is informative.

### 9.3 Conclusions

HMGB1 functions in neurodegeneration are determined by its molecular form, cellular source, and subcellular compartment. Nuclear HMGB1 contributes to chromatin organization and DNA maintenance, whereas post-translational modification, nucleocytoplasmic redistribution, and redox state regulate its release and extracellular activity. In AD, PD, and ALS models, extracellular HMGB1 participates in the propagation of inflammatory and neuronal injury. In polyglutamine and SCA models, depletion of soluble nuclear HMGB1 and impairment of intracellular maintenance functions form a distinct component of disease pathology. This compartmental framework accommodates disease-specific variation without assigning a single function to total HMGB1 across neurodegenerative disorders.

The next step is to define the temporal contribution of HMGB1 in sporadic disease, quantify the relationship between central and peripheral sources, and determine the clinical significance of its functional biochemical forms. Compartment- and cell-specific causal models, standardized redox-resolved assays, longitudinal human sampling, and interventions with documented target engagement, central nervous system exposure, and long-term safety will establish the contexts in which preserving intracellular HMGB1 or limiting extracellular signaling modifies disease course.
